# Histiocytic sarcoma simulating immune thrombocytopenic purpura

**DOI:** 10.1007/s00277-012-1606-z

**Published:** 2012-10-18

**Authors:** Joycelyn Sim, Yu-Yan Hwang, Rock Y. Y. Leung, Jason C. C. So, Yok-Lam Kwong

**Affiliations:** 1 Department of Medicine, Professorial Block, Queen Mary Hospital, Pokfulam Road, Hong Kong, China; 2 Department of Pathology, Queen Mary Hospital, Hong Kong, China

**Fig. 1:**
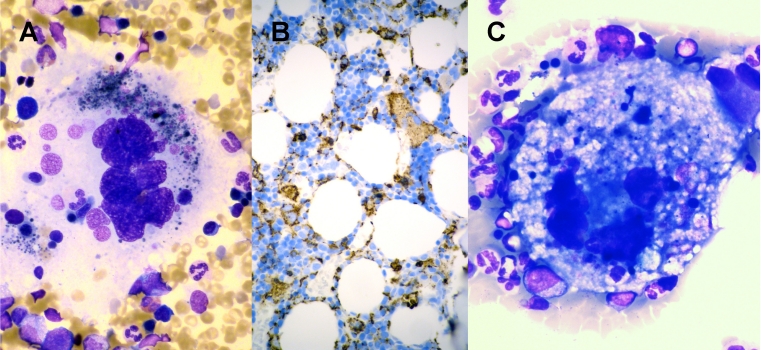
Histiocytic sarcoma infiltrating the bone marrow. **a** Histiocytic cell showing haemophagocytosis. **b** Immunohistochemical staining showing infiltration of the marrow by CD68-positive cells (immunoperoxidase). **c** Enormous histiocyte showing active haemophagocytosis

Dear Editor,

A 64-year-old woman presented with isolated thrombocytopenia and was diagnosed to have immune thrombocytopenic purpura (ITP). There was a slow but relentless deterioration of her platelet count. Two years later, she was found to have splenomegaly and a platelet count of 23 × 10^9^/L. Positron emission tomography/computed tomography showed multiple mildly hypermetabolic masses in the spleen and liver. Splenectomy and liver biopsy were performed. The spleen showed sinusoidal infiltration by large tumour cells with abundant foamy cytoplasm and active erythrophagocytosis. Tumour cells were positive for the histiocytic markers lysozyme, CD68 and CD163. Liver was also involved. Features were consistent with histiocytic sarcoma. Marrow examination showed the presence of large histiocytic cells with haemophagocytosis (Fig. 1a), which were CD68 positive (Fig. 1b). Haematopoiesis appeared otherwise normal. Her platelet count recovered briefly after splenectomy, but soon deteriorated to <10 × 10^9^/L, which was associated with frequent and distressing bleeding. Several chemotherapeutic regimens comprising cyclophosphamide, doxorubicin, vincristine, etoposide, cytarabine, bleomycin, methrotrexate, gemcitabine, cisplatin and prednisolone failed to alleviate the severe thrombocytopenia. Reassessment marrow examination showed the persistence of abnormal histiocytic cells, some reaching enormous sizes (Fig. 1c). She was finally started on the thrombopoietin mimetic eltrombopag (50 mg/day). The platelet count responded within a fortnight, rising to about 40 × 10^9^/L. Since then, she had remained asymptomatic with stable platelet counts while on eltrombopag.

This patient initially presented with what appeared to be ITP. However, the gradual deterioration and the finding of a splenomegaly prompted further investigations [1], which showed an infiltrative lesion in the spleen and liver. With the diagnosis of histiocytic sarcoma, marrow examination was performed, showing histiocytic infiltration. Therefore, the thrombocytopenia was attributed to marrow infiltration, although the extremely low platelet counts were incongruent with the preserved haematopoiesis. Intensive chemotherapy did not improve the platelet count or decrease the histiocytes. We surmised that the thrombocytopenia might instead be due to haemophagocytosis, and therefore after all was similar to ITP in that excessive destruction of platelets was the pathogenetic mechanism. She was treated with eltrombopag as for ITP refractory to splenectomy [2], and achieved a satisfactory response. Although eltrombopag cannot be expected to impact on the histiocytic sarcoma, it has significantly improved the quality of life of the patient and relieved her of severe thrombocytopenia and bleeding. Finally, although bone marrow examination may not be recommended for the majority of patients presenting with features characteristic of ITP [1], the judicious performance of this procedure may be necessary in patients with atypical features, particularly in the elderly.
